# The application value of modified thyroid imaging report and data system in diagnosing medullary thyroid carcinoma

**DOI:** 10.1002/cam4.2217

**Published:** 2019-05-09

**Authors:** Jialin Zhu, Xing Li, Xi Wei, Xueling Yang, Jing Zhao, Sheng Zhang, Zhi Guo

**Affiliations:** ^1^ Department of Ultrasound Diagnosis and Treatment, Tianjin Medical University Cancer Institute and Hospital, National Clinical Research Center of Cancer, Key Laboratory of Cancer Prevention and Therapy Tianjin's Clinical Research Center for Cancer Tianjin China; ^2^ Department of Interventional Therapy, Tianjin Medical University Cancer Institute and Hospital, National Clinical Research Center for Cancer, Key Laboratory of Cancer Prevention and Therapy Tianjin's Clinical Research Center for Cancer Tianjin China

**Keywords:** medullary thyroid carcinoma (MTC), modified, papillary thyroid carcinoma (PTC), thyroid, TI‐RADS

## Abstract

Medullary thyroid carcinoma (MTC) is highly malignant and quite different from the most common papillary thyroid carcinoma (PTC). However, most of the ultrasonic evaluation systems mainly aim at PTC at present. This study aims to evaluate the applicability of modified TI‐RADS in diagnosing MTC and compare the sonographic differences of MTC, PTC, and benign nodules. Three thousand two hundred and forty‐two thyroid nodules images confirmed by pathology were categorized according to modified TI‐RADS and ACR TI‐RADS classification. The performances of two TI‐RADS were assessed by ROC curves. The correlations between classifications with the pathology and the consistency of different doctors were evaluated. The ultrasonic differences of MTC, PTC, and benign nodules were analyzed. As a result, the number of high suspicious US features increased, the malignant risk of nodules also increased of two classifications, with significant differences between categories (*P* < 0.001). Spearman correlation coefficients were 0.751 (modified TI‐TADS) and 0.744 (ACR TI‐RADS). Areas under the ROC curve of the modified TI‐RADS and ACR TI‐RADS were 0.960 and 0.872 (*P* < 0.001). At Best cut off points, the diagnostic value of modified TI‐RADS was higher than that of ACR TI‐RADS with a higher specificity, PPV, accuracy, and Youden index). By using modified TI‐RADS to diagnose MTC and PTC, the sensitivity, specificity, NPV, accuracy, and Youden index were higher in MTC than PTC. The Kendall's correlation coefficients were 0.962, 0.930, and 0.987. MTC had special ultrasonography characters compared with PTC and benign nodules. These results suggest that modified TI‐RADS is better than ACR TI‐RADS in diagnosing thyroid carcinomas. Diagnostic value to MTC of modified TI‐RADS is slightly higher than that to PTC, and the categorical results of different doctors were consistent. MTC had several particular features contrast to PTC and benign nodules.

## 1 INTRODUCTION

Medullary thyroid carcinoma (MTC) originates from the parafollicular calcitonin secreting cells (C‐cells) with high degree malignancy. It is a rare neuroendocrine tumor and accounts for 3%‐5% of all thyroid carcinomas, but represents up to 13.4% of thyroid cancer‐related deaths.^1^, ^2^ At present, most of the classification systems mainly focus on differentiating thyroid tumors, especially on papillary thyroid carcinoma (PTC), due to its high prevalence, which accounts for more than 80% of primary thyroid malignant tumors.^3^ Contrarily, suitable classification criterion and ultrasonographic findings of MTC have rarely been reported. The biological characteristics and prognosis of MTC are remarkably different from PTC. Therefore, differentiating MTC from PTC has important clinical significance.

Ultrasonography (US) is extensively used for the preliminary evaluation of thyroid nodules, as a result of its convenience of distinguishing benign and malignant nodules. So far, several US classification systems have been proposed to standardize the evaluation of thyroid nodules. Basing on a widespread used Breast Imaging Reporting and Data System (BI‐RADS),^4^ Horvath et al and Park et al respectively proposed the Thyroid Imaging Reporting and Data System (TI‐RADS) to stratify thyroid nodules in accordance with the risk of malignancy in 2009.^5^, ^6^ However, because of the complexity of the mathematical model, they were difficult to operate well in clinical work. Modified thyroid imaging reporting and data system (modified TI‐RADS) classification was created in our institution by a multidisciplinary team and published in 2015,^7^ as an attempt to solve the problem of thyroid nodule selection for FNAB or clinical treatments. Recently, the recommendations of American College of Radiology (ACR) TI‐RADS Committee had recently been published, which provided guidance regarding treatment of thyroid nodules on the basis of ultrasound characters.^8^ In this study, we compared malignancy risk stratification and diagnostic efficiency of malignant thyroid nodules by modified TI‐RADS and ACR TI‐RADS, and evaluated the applicability of modified TI‐RADS in MTC patients compared with PTC and assessed US appearance of MTC nodules.

## 2 MATERIALS AND METHODS

### 2.1 Patients

This study included 3242 patients that received preoperative ultrasound examination and were surgically and pathologically diagnosed as MTC, PTC, nodular goiter, or adenomatous goiter in Tianjin Medical University Cancer Hospital from July 2014 to June 2017. The most suspicious nodule was selected as the object of this study in patients with multiple nodules. Among them, there were 118 MTC nodules, 1866 PTC nodules, 943 nodular goiter nodules and 315 adenomatous goiter nodules. This retrospective study was approved by the medical ethics committee of Tianjin Medical University Cancer Institute and Hospital. Written consent had been obtained from each patient after full explanation of the purpose and nature of all procedures used.

### 2.2 Procedures

US examinations were performed with a 5‐12 MHz linear array transducer (iU22; Philips Diagnostic Ultrasound System, Bothell, WA). US features of each thyroid nodule were described and recorded in our institutional database. Three doctors evaluated the characters of preoperative US images and categorized the nodules according to modified TI‐RADS and ACR TI‐RADS classification, respectively.

### 2.3 Sonographic image analysis

US features were recorded according to the following categories: size, position, composition, echogenicity, margins, calcifications, shape, vascularization, and suspicious metastatic lymph node. Composition was classified as solid (solid portion ≥90%), predominantly solid (90% >solid portion ≥50%), predominantly cystic (10%≤solid portion <50%), and cystic (solid portion <10%). The nodule was classified as hyperechoic, isoechoic, or hypoechoic to the surrounding thyroid parenchyma or marked hypoechogenicity comparing with the adjacent cervical muscle. Margins were classified as well‐ or ill‐defined (microlobulated or irregular margins). Calcifications, if present, were classified as microcalcifications or macrocalcifications (including eggshell calcifications) or mixed calcifications. Shape was classified as parallel (Wider‐than‐tall, A/T < 1) or nonparallel (taller‐than‐wide, A/T ≥ 1). Vascularization was classified as absent, few, or enhanced (compared with surrounding thyroid parenchyma). Besides, whether there were suspicious metastatic lymph nodes of thyroid origin should be checked. Abnormal appearances suggestive of cervical lymph node metastasis include a spherical shape, loss of the normal echo of lymphatic hilum, heterogeneity with cystic components, and microcalcifications.^9^, ^10^ Standardized vocabulary for ultrasonography of thyroid nodules is shown in Table 1.

**Table 1:** Standardized vocabulary for ultrasonography of thyroid nodules in modified TI‐RADS

Nodule vocabulary	Definition
Composition	Solid
	Predominantly solid
	Predominantly cystic
	Cystic
Echogenicity	Hyperechoic
	Isoechoic
	Hypoechoic
	Markedly Hypoechoic
Margins	Well‐defined
	Ill‐defined
Calcifications	None
	Macrocalcifications
	mixed calcifications
	Microcalcifications
Shape	A/T ≥ 1
	A/T < 1
Vascularization (blood flow)	Absent (avascular)
	Few
	Enhanced
Suspicious metastatic lymph node(s)	None
	One or more

Abbreviations: A/T ≥ 1, the shape of nodule is taller‐than‐wide; A/T < 1, the shape of nodule is wider‐than‐tall; TI‐RADS, Thyroid Imaging Reporting and Data System.

#### 2.3.1 Modified TI‐RADS criteria

**Figure 1:**
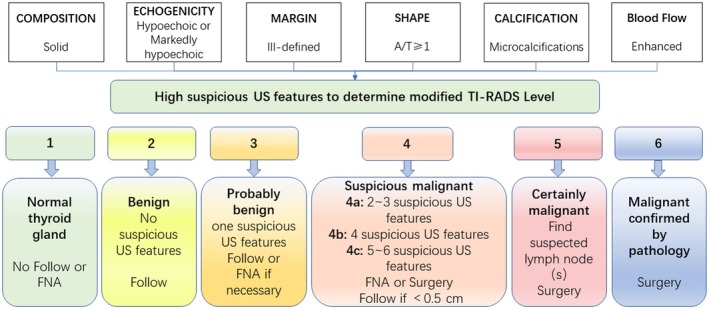
The graph showed five categories on the basis of the modified TI‐RADS vocabulary, levels, and criteria for fine‐needle aspiration, follow‐up ultrasound or surgery. TI‐RADS, Thyroid Imaging Reporting and Data System. A/T ≥ 1, the shape of nodule is taller‐than‐wide

Solid composition, marked hypoechogenicity, ill‐defined margins, microcalcifications, oval or round shape (taller‐than‐wide shape, A/T ≥ 1), and enhanced blood flow were considered high suspicious US features of malignant thyroid nodules (Figure 1). As the number of high suspicious US features increased, the risk of malignancy also increased. Level 1: normal thyroid gland, no nodule, no need to follow. Level 2: benign (risk = 0%), nodule without any of the six high suspicious US features, suggested follow‐up every year. Level 3 (risk ≤ 5%): probably benign, nodule with any one of the six high suspicious US features, suggested follow‐up every 6 months or FNAB if necessary. Level 4: suspicious malignant, 4a: nodule with any two or three of the six high suspicious US features, (risk range, 6% to 45%); 4b: nodule with any four of the six high suspicious US features, (risk range, 46% to 75%); 4c: nodule with any five or six of the six high suspicious US features, (risk range, 76% to 95%), suggested FNAB or surgery. Level 5: certainly malignant (risk > 95%), one or more suspect lymph node(s) were associated with a thyroid nodule, suggested surgery. Level 6: malignant (risk = 100%), nodule confirmed by cytology or pathology.

#### 2.3.2 ACR TI‐RADS criteria

**Figure 2:**
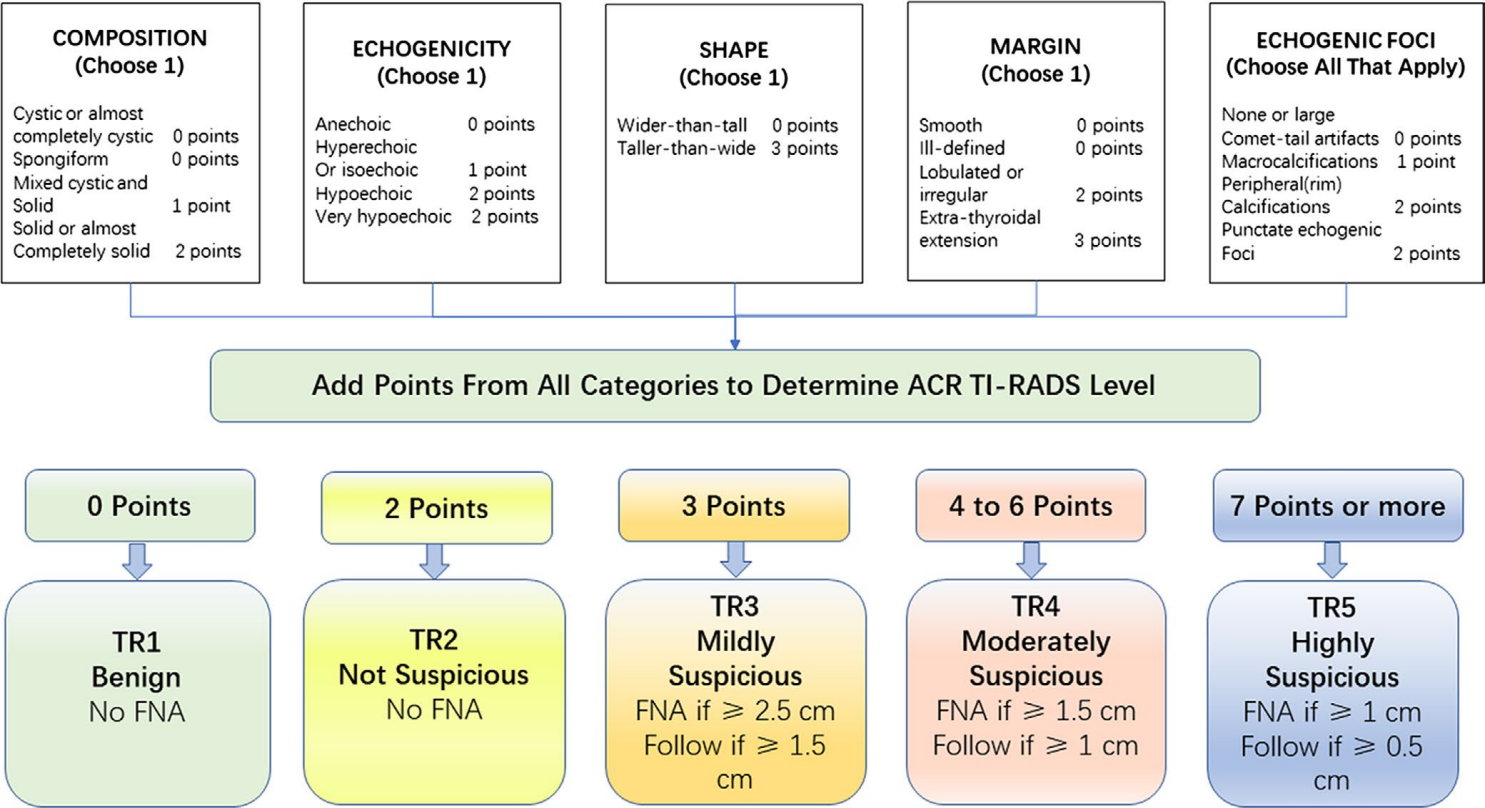
The graph showed five categories on the basis of the ACR TI‐RADS vocabulary, levels, and criteria for fine‐needle aspiration or follow‐up ultrasound. ACR, American College of Radiology; TI‐RADS, Thyroid Imaging Reporting and Data System

The ultrasound characters in the ACR TI‐RADS are categorized as benign, minimally suspicious, moderately suspicious, or highly suspicious for malignancy.^8^ Points are given for all the ultrasound features in a nodule, summing all the points one nodule contains and getting the final classification. Figure 2 presents these characters as per the five lexicon categories and detailed scoring system.

### 2.4 Receiver operating characteristic curve

Receiver operating characteristic curves were constructed to compare the differences of two TI‐RADS classifications in diagnosing malignant thyroid nodules. Also, the diagnostic value of modified TI‐RADS for MTC and PTC were evaluated by ROC curves. The sensitivity, specificity, PPV, NPV, accuracy, and Youden index were calculated at each cut off point.

### 2.5 Repeatability test

Three independent doctors having more than 5 years of experience in performing thyroid US diagnosis evaluated the characters of preoperative US images of patients and categorized the nodules by modified TI‐RADS and ACR TI‐RADS classification, respectively. The Kendall's coefficient of concordance was estimated consistency of categorizing results of different doctors.

### 2.6 Statistical analysis

Data and Statistical Analyses: Analyses were performed with software (SPSS, version 23.0; MedCalc version 18.2.1). Comparison of maximum diameter and age were analyzed using an independent sample t‐test; sonographic features, such as: position, components, echogenicity, margin, calcification, shape, blood flow, and lymph node metastasis rate were compared using χ2 test. The diagnostic efficacy of the two TI‐RADS models was assessed by estimating the area under the ROC curve, and Z statistic was used to compare the difference of the AUC. Diagnostic performances of each guideline for diagnosis of thyroid cancer were evaluated according to sensitivity, specificity, positive predictive value, negative predictive value, accuracy, and Youden index. The correlation between each classification method and pathological results was calculated by Spearman's correlation analysis. The Kendall's W test was estimated consistency of categorizing results of different doctors. All tests were two‐sided, and *P* < 0.05 was considered indicative of a statistically significant difference.

## 3 RESULTS

### 3.1 Demographic data and histological results

In this study, there were 118 MTC patients including 52 men and 66 women, 1866 PTC patients including 421 men and 1445 women, and 1258 patients with benign nodules including 263 men and 995 women. The gender differences among MTC, PTC, and benign nodules were statistically significant (*P* < 0.001). Patients with MTC were older than those with PTC (mean age, 49.19 ± 12.73 vs 42.70 ± 12.39 years, respectively; *P* < 0.001). Patients with PTC were younger than those with benign nodules (mean age, 42.70 ± 12.39 vs 49.63 ± 12.01 years, respectively; *P* < 0.001++). MTC were larger than PTC in size (1.62 ± 1.08 vs 1.11 ± 0.90 cm, respectively; *P* < 0.05). There was no statistical difference between patients with MTC and benign nodules in age and size (*P* > 0.05).

### 3.2 Comparison of modified TI‐RADS and ACR TI‐RADS scores with pathology

The malignancy rates of modified TI‐RADS category 2, 3, 4a, 4b, 4c, and 5 nodules were 0% (0 of 465 nodules), 1.49% (6/404), 60.83% (278/457), 82.02% (803/979), 94.98% (719/757), and 98.89% (178/180), respectively, with prominent differences between categories (*P* < 0.001). The malignancy rates of modified ACR category 2, 3, 4, and 5 nodules were 0% (0/269), 5.38% (22/409), 24.65% (107/434), and 87.09% (1855/2130), respectively, with prominent differences between categories (*P* < 0.001) (Table 2). The results were showed in Table 2 in detail.

**Table 2:** Modified and ACR TI‐RADS classification system in relation to pathology

	Total	Pathology		MTC	PTC	Calculated malignancy rate	Recommended malignancy risk	χ^2^	*P* value
		Benign	Malignant						
Modified TI‐RADS									
2	465	465	0	0	0	0.00%	0.00%	1990.315	<0.001
3	404	398	6	1	5	1.49%	≤5%		
4a	457	179	278	15	263	60.83%	6%‐45%		
4b	979	176	803	34	769	82.02%	46%‐75%		
4c	757	38	719	27	692	94.98%	76%‐95%		
5	180	2	178	41	137	98.89%	>95%		
Total	3242	1258	1984	118	1866				
ACR TI‐RADS									
2	269	269	0	0	0	0.00%	≤2%	279.473	<0.001
3	409	387	22	1	21	5.38%	≤5%		
4	434	327	107	6	101	24.65%	5%‐20%		
5	2130	275	1855	111	1744	87.09%	>20%		
Total	3242	1258	1984	118	1866				

Abbreviations: ACR, American College of Radiology; MTC, medullary thyroid carcinoma; PTC, papillary thyroid carcinoma; TI‐RADS, Thyroid Imaging Reporting and Data System.

### 3.3 Comparison of two TI‐RADS classification criteria for diagnosis of malignant thyroid nodules

**Figure 3:**
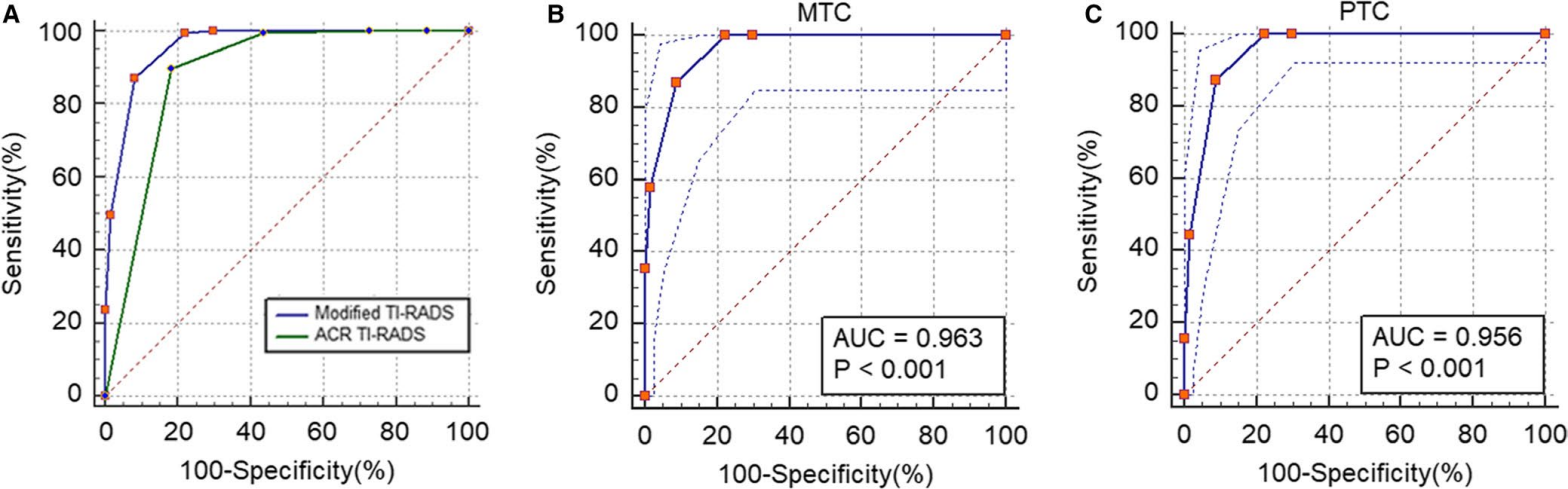
Receiver operating characteristic curve analysis. (A) Modified TI‐RADS vs ACR TI‐RADS in diagnosis of malignant nodules. Areas under the ROC curve of the modified TI‐RADS and ACR TI‐RADS were 0.960 (95% confidence interval was 0.945‐0.972) and 0.872 (95% confidence interval was 0.860‐0.883), and the difference between areas was 0.101 (95% confidence interval was 0.0911‐0.112, *P* < 0.0001). (B) Modified TI‐RADS in diagnosis of MTC nodules. Areas under the ROC curve of the modified TI‐RADS in diagnosing of MTC were 0.963 (95% confidence interval was 0.930 to 0.984). (C) Modified TI‐RADS in diagnosis of PTC nodules. Areas under the ROC curve of the modified TI‐RADS in diagnosing of PTC were 0.956 (95% confidence interval was 0.924‐0.978). MTC, medullary thyroid carcinoma; PTC, papillary thyroid carcinoma; ACR, American College of Radiology; TI‐RADS, Thyroid Imaging Reporting and Data System

Areas under the ROC curve of the modified TI‐RADS and ACR TI‐RADS were 0.960 (95% confidence interval was 0.945‐0.972) and 0.872 (95% confidence interval was 0.860‐0.883), and the difference between areas was 0.101 (95% confidence interval was 0.0911‐0.112, *P* < 0.0001) (Figure 3A). Best cut‐off point for diagnosing malignant by modified TI‐RADS and ACR TI‐RADS were ≥4a and ≥5, and at that point, diagnostic value of modified TI‐RADS were higher than ACR TI‐RADS (sensitivity, 83.35% vs 87.09%; specificity, 99.31% vs 88.40%; PPV, 99.70% vs 93.50%; NPV 68.60% vs 78.14%; accuracy, 87.63% vs 87.54%; and Youden index, 0.83 vs 0.76) (Table 3).

**Table 3:** Sensitivity, specificity, positive predictive value (PPV), negative predictive value (NPV), accuracy, and Youden index for malignant pathology of the modified and ACR TI‐RADS categories

	Sensitivity, %	Specificity, %	PPV, %	NPV, %	Accuracy, %	Youden index
Modified TI‐RADS						
3	71.44	100.00	100.00	36.96	75.54	0.714
4a	83.35	99.31	99.70	68.60	87.63	0.827
4b	88.73	78.58	85.69	82.83	84.58	0.673
4c	95.73	52.84	45.21	96.82	65.24	0.486
5	98.89	41.02	8.97	99.84	44.23	0.399
ACR TI‐RADS						
3	66.73	100.00	100.00	21.38	69.49	0.667
4	76.52	96.76	98.89	52.15	80.75	0.733
5	87.09	88.40	93.50	78.14	87.54	0.755

Abbreviations: ACR, American College of Radiology; TI‐RADS, Thyroid Imaging Reporting and Data System.

### 3.4 Comparison of diagnostic efficacy of modified TI‐RADS for PTC and MTC

Areas under the ROC curve of the modified TI‐RADS in MTC and PTC were 0.963 (95% confidence interval was 0.930 to 0.984) and 0.956 (95% confidence interval was 0.924‐0.978) (Figure 3B,3). Best cut‐off point for diagnosing MTC and PTC by modified TI‐RADS were ≥5 and ≥5, and at that point, diagnostic value of MTC were higher than PTC by using modified TI‐RADS (sensitivity, 95.35% vs 82.49%; specificity, 94.22% vs 99.42%; PPV, 34.75% vs 99.73%; NPV 99.84% vs 68.60%; accuracy, 94.26% vs 87.20%, and Youden index, 0.90 vs 0.82) (Table 4).

**Table 4:** Sensitivity, specificity, positive predictive value (PPV), negative predictive value (NPV), accuracy and Youden index for MTC and PTC of the modified TI‐RADS categories

	Sensitivity, %	Specificity, %	PPV, %	NPV, %	Accuracy, %	Youden index
MTC						
4b	32.08	98.49	86.44	82.83	83.14	0.306
4c	62.96	96.06	57.63	96.82	93.46	0.590
5	95.35	94.22	34.75	99.84	94.26	0.896
PTC						
3	70.18	100.00	100.00	36.96	74.62	0.702
4a	82.49	99.42	99.73	68.60	87.20	0.819
4b	88.09	79.54	85.64	82.83	84.51	0.676
4c	95.40	54.01	44.43	96.82	65.52	0.494
5	98.56	42.08	7.34	99.84	44.59	0.406

Abbreviations: MTC, medullary thyroid carcinoma; PTC, papillary thyroid carcinoma; TI‐RADS, Thyroid Imaging Reporting and Data System.

### 3.5 Evaluation of modified TI‐RADS

There were significant differences inside each category respectively (*P* < 0.001). Spearman's rank correlation coefficient of modified TI‐RADS and pathology was 0.751, and that of ACR TI‐RADS and pathology was 0.744. The Kendall's coefficients of concordance of categorizing results of different doctors were 0.962, 0.930, 0.987 separately, and the correlation was significant at the level of 0.01 (double tail).

### 3.6 Comparative analysis of ultrasound characteristics of MTC and PTC

**Figure 4:**
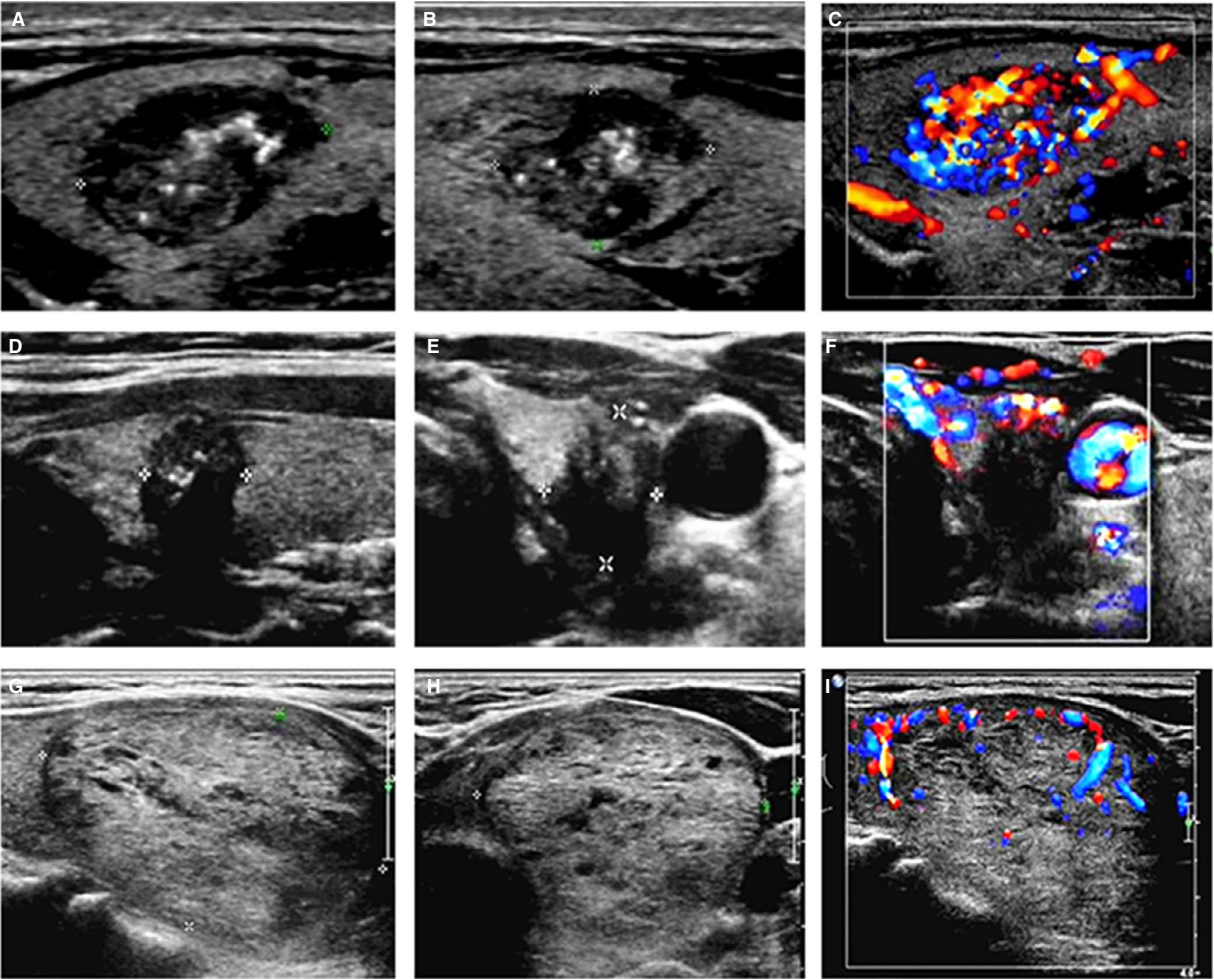
Ultrasonic images of different kinds of thyroid nodules. (A‐C) were ultrasound images of a case of MTC. Male patient, 49 years old. (A) Lesion was solid, markedly hypoechoic, well‐defined, with microcalcification. (B) A/T < 1. C. Enhanced blood flow. (D‐F) were ultrasound images of a case of PTC. Female patient, 47 years old. (D) Lesion was solid, markedly hypoechoic, with microcalcification, ill‐define. (E) A/T ≥ 1. (F) Absent of blood flow. (G‐I) were ultrasound images of a case of benign nodules. Female patient, 63 years old. (G) Lesion was almost solid, isoechoic, well‐defined, with none of calcifications. (H) A/T < 1. (I) Few blood flow. MTC, medullary thyroid carcinoma; PTC, papillary thyroid carcinoma; A/T ≥ 1, the shape of nodule is taller‐than‐wide; A/T < 1, the shape of nodule is wider‐than‐tall

The difference in position, echogenicity, margins, shape, vascularization, and whether existing suspicious metastatic lymph node of MTC with PTC was statistically significant, whereas, the difference in composition and calcifications had no statistical significance. Most of the lesions in MTC were located in the middle or upper thyroid (85.59%, 101/118), easy to show markedly hypoechoic (27.97%, 33/118), well‐defined (36.44%, 43/118) (Figure 4A) and oval‐shaped (53.39%, 63/118) (Figure 4B) comparing with PTC. The 31.36% of MTC had enhanced blood flow signals (Figure 4C), while in most of PTC, blood flow was absent (68.97%, 1287/1866, *P* < 0.001) (Figure 4F). Occurrence rate of metastatic lymph node was higher than the PTC group (34.75% vs 7.34%, *P* < 0.001). The details were shown in Table 5.

**Table 5:** Comparative analysis of ultrasound characteristics of MTC and PTC

US features	MTC (*n* = 118)	PTC (*n* = 1866)	χ^2^	*P* value
Position				
Upper	55 (46.61%)	647 (34.67%)	29.316	<0.001
Middle	46 (38.98%)	488 (26.15%)		
Down	17 (14.41%)	731 (39.17%)		
Composition				
Solid	114 (96.61%)	1775 (95.14%)	1.242	0.537
Predominantly solid	4 (3.39%)	73 (3.91%)		
Predominantly cystic	0 (0.00%)	18 (9.64%)		
Cystic	0 (0.00%)	0 (0.00%)		
Echogenicity				
Markedly hypoechoic	33 (27.97%)	477 (25.56%)	10.561	0.014
Hypoechoic	78 (66.10%)	1344 (72.03%)		
Isoechoic	7 (5.93%)	34 (1.82%)		
Hyperechoic	0 (0.00%)	11 (0.59%)		
Margin				
Well‐defined	43 (36.44%)	306 (16.41%)	30.752	<0.001
Ill‐defined	75 (63.56%)	1560 (83.59%)		
Margin				
≥1	55 (46.61%)	1316 (70.52%)	92.845	<0.001
<1	63 (53.39%)	550 (29.48%)		
Calcifications				
None	36 (30.51%)	544 (29.18%)	4.436	0.218
Microcalcifications	53 (44.92%)	953 (51.06%)		
Macrocalcifications	12 (10.17%)	108 (5.78%)		
Mixed calcifications	17 (14.41%)	261 (13.98%)		
Vascularization				
Absent (avascular)	33 (27.97%)	1287 (68.97%)	93.899	<0.001
Few	48 (40.68%)	398 (21.33%)		
Enhanced	37 (31.36%)	181 (9.70%)		
Suspicious metastatic lymph node(s)				
One or more	41 (34.75%)	137 (7.34%)	102.052	<0.001
None	77 (65.25%)	1729 (92.66%)		

Abbreviations: A/T ≥ 1, the shape of nodule is taller‐than‐wide; A/T < 1, the shape of nodule is wider‐than‐tall; MTC, medullary thyroid carcinoma; PTC, papillary thyroid carcinoma.

### 3.7 Comparative analysis of ultrasound characteristics of MTC and benign thyroid nodules

Comparing with benign thyroid nodules, MTC showed significant difference in all ultrasonographic features (*P* < 0.05), including solid (114/118, 96.61%), hypoechoic or marked hypoechoic (111/118, 94.07%), ill‐defined margin (75/118, 63.56%), microcalcifications (53/118, 44.92%), enhanced blood flow (37/118, 31.36%), and metastatic cervical lymph node (41/118, 34.74%). The details were shown in Table 6.

**Table 6:** Comparative analysis of ultrasound characteristics of the MTC and benign thyroid nodules

US features	MTC (*n* = 118)	Benign nodules (*n* = 1258)	χ^2^	*P* value
Position				
Upper	55 (46.61%)	397 (31.54%)	34.978	<0.001
Middle	46 (38.98%)	329 (26.14%)		
Down	17 (14.41%)	532 (42.32%)		
Composition				
Solid	114 (96.61%)	570 (45.28%)	113.684	<0.001
Predominantly solid	4 (3.39%)	493 (39.22%)		
Predominantly cystic	0 (0.00%)	86 (6.87%)		
Cystic	0 (0.00%)	109 (8.63%)		
Echogenicity				
Markedly hypoechoic	33 (27.97%)	186 (14.82%)	29.399	<0.001
Hypoechoic	78 (66.10%)	765 (60.78%)		
Isoechoic	7 (5.93%)	202 (16.04%)		
Hyperechoic	0 (0.00%)	105 (8.36%)		
Margin				
Well‐defined	43 (36.44%)	1048 (83.29%)	144.288	<0.001
Ill‐defined	75 (63.56%)	210 (16.71%)		
Shape (A/T)				
≥1	55 (46.61%)	239 (19.00%)		
<1	63 (53.39%)	1019 (81.00%)		
Calcifications				
None	36 (30.51%)	740 (58.82%)	128.626	<0.001
Microcalcifications	53 (44.92%)	141 (11.21%)		
Macrocalcifications	12 (10.17%)	314 (24.96%)		
Mixed calcifications	17 (14.41%)	63 (5.01%)		
Vascularization				
Absent (avascular)	33 (27.97%)	502 (39.90%)	31.930	0.001
Few	48 (40.68%)	598 (47.54%)		
Enhanced	37 (31.36%)	158 (12.56)		
Suspicious metastatic lymph node(s)				
One or more	41 (34.75%)	2 (0.16%)	426.288	<0.001
None	77 (65.25%)	1256 (99.84%)		

Abbreviations: MTC, medullary thyroid carcinoma; A/T ≥ 1, the shape of nodule is taller‐than‐wide; A/T < 1, the shape of nodule is wider‐than‐tall.

## 4 DISCUSSION

Several TI‐RADS have been developed for malignancy risk stratification that incorporate US features to categorize thyroid nodules and recommend FNAB. In 2009, Horvath et al firstly used suspicious US features and associated the risk of malignant tumors with the number of suspected ultrasound signs,^5^ but category 4B was associated with a wide range of malignancy risk (10%‐80%), which was quite difficult to distinguish benign and malignant nodules. This problem also existed in classification of Kwak et al,^11^ in which category 4c was associated with a wide range of fitted probability of malignancy (21.0%‐91.9%). Park et al proposed an equation for predicting the probability of malignancy based on 12 US features,^6^ but it was complicated to assign each thyroid nodule to a proposed equation in clinical practice. Recently, ACR TI‐RADS ultrasound classifications had been released,^8^ providing guidance for management of thyroid nodules bace on ultrasound features, summing the points and stratified the risk of malignancy into five categories. However, category 5 (highly suspicious), allocated a median score of 7 or more, comprised a relatively low malignancy risk and a wide range of malignancy probability (>20%), and it was a little complex to calculate all points from all categories.

To overcome the associated complexity, Zhang and his team developed modified TI‐RADS model that classifies nodules according to only six high suspicious US features, which permitted rapid calculation and immediate estimation of the possible malignancy risk. Because of it was simple to count the number of suspicious US features and got a modified TI‐RADS level, it was feasible to be applied to clinical practice. We found that as the number of high suspicious US features increased, the modified TI‐RADS level and malignant risk of a nodule also increased, which was consistent with the result of Wang et al.^7^ In this research, the malignancy rates of TI‐RADS category 2, 3, 4a, 4b, 4c, and 5 nodules were 0, 1.49% (6/404), 60.83% (278/457), 82.02% (803/979), 94.98% (719/757), and 98.89% (178/180), respectively. In this study, nodules of 4a and 4b had higher risks of malignancy than the recommended value. It might because that parts of patients with these grades of nodule(s) chosen to be followed up rather than surgical treatment, which may lead to a degree of statistical bias. The difference between each level was statistically significant, which proved that modified TI‐RADS could perfectly classify thyroid nodules with different malignant risks. Based on this, different recommendations were put forward for different levels of nodules, providing appropriate advice to clinicians.

Both modified TI‐RADS and ACR TI‐RADS proved to be impactful methods in diagnosing malignant thyroid nodules in clinic with a high value of area under the ROC curve (0.960, 0.872) and Spearman's rank correlation coefficient (0.751, 0.744), with pathology as the gold standard. A study of Lauria et al indicated that the ACR TI‐RADS classification has the highest area under the ROC curve for the identification of cytological high‐risk nodules in comparison with the other two classifications.^12^ In our result, the area under the ROC curve of modified TI‐RADS was significantly higher than ACR TI‐RADS for diagnosing malignant thyroid nodules confirmed by postoperative pathology. Kendall's coefficients of concordances proved that different doctors showed excellent agreements with each other by using modified TI‐RADS in diagnosis of thyroid carcinoma. The discrimination ability of modified TI‐RADS was significantly higher than that of ACR TI‐RADS. At the best cut off points for diagnosing malignant nodules by modified TI‐RADS and ACR TI‐RADS, the specificity, PPV, accuracy, and Youden index were higher than of ACR TI‐RADS. When using modified TI‐RADS to diagnose MTC and PTC, the sensitivity, specificity, NPV, accuracy, and Youden index were higher in MTC (cut off point = 5) comparing to PTC (cut off point = 4a). This may be due to the involvement of vascularization and suspicious metastatic lymph node in the design of modified TI‐RADS, and abundant blood flow and high metastasis rate were special characters of MTC.^13^

In this study, MTC occurred in older patients (49.19 years ± 12.73 vs 42.70 years ± 12.39, *P* < 0.001) and had larger sizes (1.62cm ± 1.08 vs 1.11cm ± 0.90, *P* < 0.05) compared with PTC. Incidence of MTC in males and females was similar (52 vs 66) while PTC and benign nodules were more common in women than men (421 vs 1445), which were consistent with the findings of Wolinski et al and Liu et al.^14^, ^15^ MTC mostly located in the middle or upper parts of the thyroid gland (85.58%, 101/118). Because MTC originates from the parafollicular C‐cells which are mostly distributed in the middle and upper part of the thyroid gland.^16^

MTC is a kind of tumor with high degree of malignancy, and has obvious characteristics of malignant tumor. In this study, MTC had significant malignant US features compared with benign nodules. (1) MTC was mostly solid (114/118), which was mainly related to the rich blood supply, and not easy to occur liquefaction and necrosis. (2) MTC usually showed hypoechoic (77/118) or markedly hypoechoic (33/118), relating to a large number of collagen fiber organization, less ground substance and abundant blood vessels in pathology.^17^ (3) The margin of MTC was relatively ill‐defined, accounting for 63.56% (75/118), while the margin of benign nodules was smooth (83.29%, 1048/1258) (Figure 4G). Whether the boundary was clear or not depended on the type of invasion. MTC had no capsule, infiltrated and grew extensively, so the boundary was not clear, and it was easy to invade the thyroid envelope and metastasize to adjacent lymph nodes. (4) Calcification was common in MTC, of which microcalcification accounts for 44.92% (53/118), and this result was similar with Yun et al,^18^ while microcalcification in benign nodular was rare, accounting for 11.21% (141/1258). Microcalcification was a specific manifestation of thyroid cancer. Due to the rapid growth of malignant tumor cells and excessive proliferation of blood vessels and fibrous tissues in the tumor, calcium salt deposition were prone to occur, or the tumor itself secretes some substances, which lead to calcification.^19^ Microcalcification need to be distinguished from hyperechogenic crystals formed by concentrated colloids in thyroid nodules, which was usually with comet tail signs behind the crystals and it thought to be a benign ultrasonic sign.

Meanwhile, MTC was a special type of the thyroid carcinoma, which was significantly different from the most common PTC. In this study, MTC had plenty of specific US features: (1) The shape of MTC nodules was easy to manifest as round or oval (63/118 vs 550/1866, *P* < 0.001) comparing with PTC. This was mainly related to the abundant blood flow and easy growth of MTC. (2) The margin of MTC was relatively well‐defined, and mainly due to its compression of normal thyroid tissue around the tumor and the formation of pseudo capsule. This result was consistent with those of most scholars.^20^, ^21^ (3) There was no difference in calcification between MTC and PTC in US performance. However, in pathology, the calcification of PTC was psammoma body,^22^ while the calcification of MTC was mostly amyloid‐like content deposited in the intercellular substance (11). (4) MTC was easier to appear metastasis than PTC. It was reported that 70% of patients with MTC who presented with a palpable thyroid nodule had cervical metastasis and 10% had distant metastasis.^23^ In this research, the lymph node metastasis rate of MTC was 34.75% (41/118), which was significantly higher than that of PTC (7.34%, 137/1866). As tumor staging was an important factor affecting the prognosis of MTC patients, improving the accuracy of early diagnosis plays an important role in the prognosis.

Medullary thyroid carcinoma is a neuroendocrine tumor and the most significant clinical feature is the elevation of calcitonin and carcino‐embryonic antigen (CEA).^24^, ^25^ Calcitonin and CEA are the most valuable tumor markers of MTC, and serum concentration of them is directly related to the number of c‐cells. Therefore, it is necessary to examine serum calcitonin and CEA in patients who are highly suspected to be MTC. In addition, ultrasound‐guided fine needle aspiration biopsy (FNAB) is one of the most useful and accurate methods in the diagnosis of thyroid carcinoma with good sensitivity and specificity and optimal concordance to the final postoperative pathology.^26^, ^27^ FNA is also an approved modality for the diagnosis of MTC with high sensitivity.^28^, ^29^ Dyhdalo et al reported that in 61 cases of MTC that had both a preoperative FNAB and a thyroidectomy, MTC was diagnosed correctly in 86% (44/ 51).^30^ However, a study indicated that fine‐needle aspiration cytology (FNAC) was able to detect ~50% of MTC lesions, suggesting that other techniques may be needed in combination with FNAC to diagnose MTC and avoid false negative result.^31^ Some researches demonstrated that calcitonin measurement in washout fluids of needle after aspiration had higher sensitivity than cytology in diagnosing MTC.^32^, ^33^

In conclusion, modified TI‐RADS could provide helpful guidelines in deciding the optimal strategies for management of thyroid nodules, which was better than ACR TI‐RADS in several respects. The value of modified TI‐RADS for diagnosis of MTC is higher than that of PTC. MTC had plenty of specific US features contrast to PTC, such as locating in middle and upper parts of the thyroid, marked hypoechogenicity, relatively smooth margin, oval or round shape, abundant blood flow, and high rate of lymph node metastasis.

## CONFLICT OF INTEREST

The authors declare no conflict of interest.

## AUTHOR CONTRIBUTIONS

Zhi Guo, Sheng Zhang and Jialin Zhu had made substantial contributions to conception and design; Jialin Zhu, Xing Li, Xueling Yang, Xi Wei, and Jing Zhao had made substantial contributions to acquisition of data, or analysis and interpretation of data; Jialin Zhu and Xing Li were involved in drafting the manuscript or revising it critically for important intellectual content.
